# Howler monkey foraging ecology suggests convergent evolution of routine trichromacy as an adaptation for folivory

**DOI:** 10.1002/ece3.2716

**Published:** 2017-02-04

**Authors:** Amanda D. Melin, Vishal Khetpal, Yuka Matsushita, Kaile Zhou, Fernando A. Campos, Barbara Welker, Shoji Kawamura

**Affiliations:** ^1^ Department of Anthropology and ArchaeologyUniversity of CalgaryCalgaryABCanada; ^2^ Department of Medical Genetics and Alberta Children’s Hospital Research InstituteUniversity of CalgaryCalgaryABCanada; ^3^ Department of AnthropologyWashington University in St. LouisSt. LouisMOUSA; ^4^ Department of Integrated BiosciencesGraduate School of Frontier SciencesThe University of TokyoTokyoJapan; ^5^ Department of Plant ProtectionCollege of Agriculture and BiotechnologyZhejiang UniversityHangzhouZhejiangChina; ^6^ Department of AnthropologyTulane UniversityNew OrleansLAUSA; ^7^ Department of AnthropologyState University of New York at GeneseoGeneseoNYUSA

**Keywords:** *Alouatta*, color vision, *Ficus*, molecular evolution, opsin, polymorphism, primate evolution, sensory ecology

## Abstract

Primates possess remarkably variable color vision, and the ecological and social factors shaping this variation remain heavily debated. Here, we test whether central tenants of the folivory hypothesis of routine trichromacy hold for the foraging ecology of howler monkeys. Howler monkeys (genus *Alouatta*) and paleotropical primates (Parvorder: Catarrhini) have independently acquired routine trichromacy through fixation of distinct mid‐ to long‐wavelength‐sensitive (*M/LWS*) opsin genes on the X‐chromosome. The presence of routine trichromacy in howlers, while other diurnal neotropical monkeys (Platyrrhini) possess polymorphic trichromacy, is poorly understood. A selective force proposed to explain the evolution of routine trichromacy in catarrhines—reliance on young, red leaves—has received scant attention in howlers, a gap we fill in this study. We recorded diet, sequenced *M/LWS* opsin genes in four social groups of *Alouatta palliata,* and conducted colorimetric analysis of leaves consumed in Sector Santa Rosa, Costa Rica. For a majority of food species, including *Ficus* trees, an important resource year‐round, young leaves were more chromatically conspicuous from mature leaves to trichromatic than to hypothetical dichromatic phenotypes. We found that 18% of opsin genes were *MWS*/*LWS* hybrids; when combined with previous research, the incidence of hybrid *M/LWS* opsins in this species is 13%. In visual models of food discrimination ability, the hybrid trichromatic phenotype performed slightly poorer than normal trichromacy, but substantially better than dichromacy. Our results provide support for the folivory hypothesis of routine trichromacy. Similar ecological pressures, that is, the search for young, reddish leaves, may have driven the independent evolution of routine trichromacy in primates on separate continents. We discuss our results in the context of balancing selection acting on New World monkey opsin genes and hypothesize that howlers experience stronger selection against dichromatic phenotypes than other sympatric species, which rely more heavily on cryptic foods.

## 1 Introduction

Color vision is an ancient and plastic trait of many vertebrate and arthropod animals that can reveal adaptations to diet, social communication, and the environment (e.g., Bennett & Thery, 2007; Johnsen et al., 2006; Osorio & Vorobyev, 2008). Tetrachromatic color vision based on four opsin genes is common among diurnal, terrestrial birds and reptiles. However, the majority of placental mammals possess dichromatic vision due to loss of two opsin genes, which has been linked to extended periods of nocturnality in their evolution (Bowmaker, 1998; Jacobs & Rowe, 2004). The ability of primates to make color discriminations is heightened compared to most mammals; yet, color vision among primate taxa is surprisingly variable.

Among Old World monkeys, apes, and humans (parvorder: Catarrhini), both males and females discern hues in the red‐green range of the visual spectrum, in addition to hues along the ancestral blue‐yellow color axis (Jacobs, 1996). This “routine” trichromacy is enabled by two distinct opsin genes on the X‐chromosome, which code for mid‐wavelength‐sensitive (MWS, green) and long‐wavelength‐sensitive (LWS, red) cone photopigments, and a third autosomal opsin gene, which codes for short‐wavelength‐sensitive (SWS, blue) pigments. In contrast, some lemurs and most New World primate species possess polymorphic trichromacy due to allelic variation of a single mid‐ to long‐wavelength‐sensitive opsin gene (*M/LWS*) (reviewed in Kawamura et al., 2012). In these polymorphic species, some females and all males are red‐green color‐blind, and only females that are *M/LWS* heterozygotes are capable of trichromatic vision (Jacobs, 1996; Valenta et al., 2015; Veilleux & Bolnick, 2009). Howler monkeys (genus: *Alouatta*), however, are an exception to this pattern and have independently evolved routine trichromacy akin to that possessed by catarrhines (Dulai, von Dornum, Mollon, & Hunt, 1999; Jacobs, Neitz, Deegan, & Neitz, 1996). *Alouatta* is therefore an ideal model for testing competing hypotheses on the adaptive origins of trichromatic color vision.

A prevalent practice among researchers studying primate color vision has been to seek a unifying selective agent regardless of whether the expression of trichromatic vision is routine or polymorphic in a species. Revisiting formal hypotheses highlights a subtle distinction that was often overlooked in ensuing studies: polymorphic trichromatic vision was tied to efficient detection of both ripe fruits and cryptic foods, including fruits and insects (Dominy, Svenning, & Li, 2003; Riba‐Hernández, Stoner, & Lucas, 2005 p.; Melin, Fedigan, Hiramatsu, Sendall, & Kawamura, 2007; Melin, Hiramatsu et al., 2014), whereas routine trichromatic vision was tied to either consumption of reddish‐yellowish fruits or seasonal young leaf consumption (Dominy & Lucas, 2001, 2004; Regan et al., 2001; Sumner & Mollon, 2000a,b). To conflate this distinction and assume identical selective pressures for different visual systems may be stalling progress as different evolutionary mechanisms could be at play. To understand the evolution of routine versus polymorphic trichromacy, it is essential to have independent systems with which to test predictions of previous hypotheses. *Alouatta* provides such an opportunity for testing the folivory hypothesis of routine trichromacy, which was developed based on studies of catarrhine foraging ecology and food properties (Dominy & Lucas, 2004; Lucas et al., 2003).

**Figure 1:**
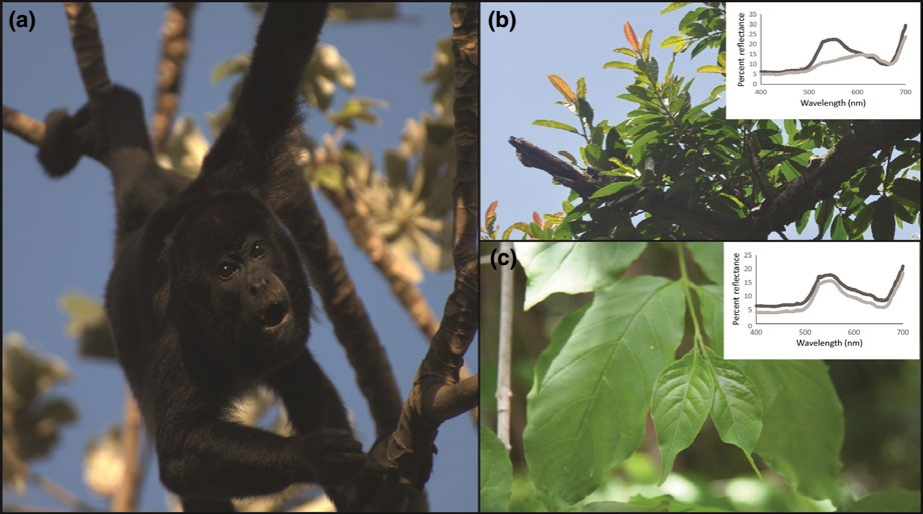
Study subject and representative dietary items. An adult male howler monkey (*Alouatta palliata*) forages on leaves in Sector Santa Rosa, Área de Conservación Guanacaste (a). Like other member of this genus, both male and female mantled howler monkeys possess routine trichromacy based on a short‐wavelength (bluish)‐sensitive opsin and distinct mid (greenish)‐ and long (reddish)‐wavelength‐sensitive photopigments. Reflectance spectra and corresponding photographs of dietary plants consumed by howler monkeys in Sector Santa Rosa, Costa Rica show color variation in accordance with phenophase (b,c). Some species, including *Ficus bullenoi* (b), have immature leaves that change color from reddish to greenish as they mature. *Exostema mexicanum* (c) exemplifies species whose immature leaves (smaller leaves at terminus of branch) are similar in chromaticity to mature leaves. Reflectance spectra (insets) quantify mean chroma of immature leaves (light gray data points, *n* = 5 leaves) and mature leaves (dark gray data points, *n* = 5 leaves) along the visible spectrum (400–700 nm). Photographs were taken in Sector Santa Rosa by AD Melin (a,b) and A. Guadamuz (c)

In general, platyrrhines are smaller than catarrhines, and for many species, leaves do not comprise a substantial proportion of the diet (Campbell, Fuentes, MacKinnon, Bearder, & Stumpf, 2011). The genus *Alouatta*, however, contains among the largest neotropical monkeys and also the most folivorous (Milton & McBee, 1983; Rosenberger, Halenar, & Cooke, 2011; Espinosa‐Gómez, Gómez‐Rosales, Wallis, Canales‐Espinosa, & Hernández‐Salazar, 2013; Figure 1). Like other platyrrhines, howlers consume large amounts of fruit and, when available, they often prefer fruits to leaves (Behie & Pavelka, 2015; Welker, 2004); however, fruit abundance varies seasonally and while many New World monkeys supplement frugivorous diets with insects, leaves can comprise up to 100% of howler diets (Milton, 1980). It is plausible, then, that the evolution of routine trichromacy in howler monkeys represents an adaptation for folivory. Howler monkeys are known to consume both fruits and leaves with chromaticities that would be more visible to trichromats than to dichromats (Regan et al., 1998; Lucas et al., 2003; Figure 1). However, previous studies have not tested crucial predictions of the folivory hypothesis for routine trichromatic vision. For example, the study by Lucas et al. (2003) did not account for seasonal variation in leaf availability or color. Furthermore, these studies predated the verification and discovery of anomalous trichromatic vision in *Alouatta*. Recently, a recombinant gene, intermediate to *MWS* and *LWS* opsins, was found in Costa Rican mantled howlers (*Alouatta palliata;* Gray, 1849), and a distinct recombinant *M/LWS* opsin gene was found in Yucatan black howlers (*A. pigra*, Gray, 1849) in Belize (Matsushita, Oota, Welker, Pavelka, & Kawamura, 2014). However, the consequences of this opsin variation in the context of foraging have yet to be examined.

Here, we focus on *A. palliata* and allied taxa to explore the functional significance of routine, anomalous trichromatic vision with a focus on the seasonal availability of young leaves to test whether this food is indeed a temporally critical food. We ask the following research questions: (1) Are the young leaves of dietary species chromatically distinct from mature leaves in wavelengths that are more visible to trichromatic than to hypothetical dichromatic phenotypes? If so, what is the importance of these species in the howler diet? This research will test whether the central tenant of the folivory hypotheses of routine trichromacy—that is seasonal reliance on young leaves that are reddish in coloration—holds in the Neotropics. Alternatively, if young leaves are not reddish or more conspicuous to trichromats, it would suggest that the advantages to routine trichromacy lie elsewhere or that howlers are the only platyrrhine lineage to have experienced neutral spread of an occasional opsin duplication event. (2) How frequent are standard versus anomalous genotypes of M/LWS opsin in this study population of mantled howler monkeys? By extending the survey of *MWS* and *LWS* opsin genes of Costa Rican mantled howlers, we will better understand the genetic underpinnings and variability of trichromacy in this species. (3) How does the estimated visual performance of howler monkeys with anomalous trichromacy compare, relative to the standard trichromatic phenotype? Through this line of inquiry, we will better understand the functional consequences of opsin gene variation, and assess the potential impact of genetic hybridization on the foraging performance of howlers. 4) What is the extent of seasonal variation in young leaf flush among tree species in howler monkey habitats? With this information, we can begin to assess the seasonal importance of young leaves, and how this relates to other dietary foods.

## 2 Materials and Methods

### 2.1 Study site and subjects

We conducted our study in the tropical dry forest of Sector Santa Rosa (SSR), Área de Conservación Guanacaste (ÁCG), in northwestern Costa Rica (10°45′ to 11°00′N and 85°30′ to 85°45′W). Tropical dry forests are a critically endangered biome that experience strong seasonal variation in rainfall and temperature (Campos, Jack, & Fedigan, 2015; Janzen, 1988). The dry season at SSR typically spans late November into early May, with high daily temperatures and prolonged dry conditions in April and early May; the temperature drops with the onset of the rainfall in mid‐May (Campos & Fedigan, 2009; Melin, Young, Mosdossy, & Fedigan, 2014). Three sympatric primates inhabit SSR: the white‐faced capuchin monkey, *Cebus capucinus imitator*, the black‐handed spider monkey, *Ateles geoffroyi*, and the mantled howler monkey, *Alouatta palliata*.

Mantled howler monkeys inhabit large portions of Central and South America. In Sector Santa Rosa, mean group size is typically between ten and twenty, but groups range from two to thirty individuals (Fedigan & Jack, 2012). They are sexually dimorphic in body size; males range from 4.5 to 9.8 kg and females from 3.1 to 7.6 kg; however, *A. palliata* tends to have a lower degree of sexual dimorphism than other *Alouatta* species (Campbell et al., 2011; Estrada, Garber, Pavelka, & Luecke, 2006). Mantled howlers are selective foragers, and when young leaves are present, they are preferentially consumed over mature leaves, typically due to higher nutritive value and lower concentrations of mechanical or chemical feeding deterrents in immature leaves (Milton, 1980). Howlers also consume higher energy food sources when available, particularly seasonal fruit, although they often face heavy competition from dominant, sympatric monkey species when foraging in fruiting trees (Cristóbal‐Azkarate & Arroyo‐Rodríguez, 2007).

### 2.2 Sampling genomic DNA from feces

We collected 111 fresh fecal samples from four social groups (CG, BH, SE, and CJ) of mantled howler monkeys from 2009 to 2013. Group size ranged from 7 (SE) to 13 (CG). Two females from CG group have previously been analyzed (Matsushita et al., 2014). It was not possible to match fecal samples to individual monkeys, as our sample collection did not coincide with behavioral observations. However, we made efforts to sample each group in a manner that minimized repetition of the same monkeys and maximized the number of different individuals sampled. We collected feces only when we witnessed defecation events, and when we were able to recognize that the samples were from different individuals of known sex. We extracted DNA from all fecal samples in a biological safety cabinet using QIAamp DNA Stool Mini Kit (Qiagen, Crawley, UK). The content of genomic DNA derived from the monkeys in the fecal DNA was quantified by real‐time PCR (Hiramatsu et al., 2005).

### 2.3 Color vision genotyping and determination of the anomalous trichromatic phenotype

We conducted genotyping of the *MWS* and *LWS* opsin genes for a subset (12 samples from 12 individuals) wherein the monkey genomic DNA concentration was higher than 200 pg/L (Hiramatsu et al., 2005). According to the “three‐sites” rule, amino acid changes from *LWS* to *MWS* at the three sites, (1) Ser to Ala at site 180 (_Ser_180_Ala_) on exon 3, along with (2) _Tyr_277_Phe_, and (3) _Thr_285_Ala_ on exon 5, are expected to cause −5‐, −10‐, and −17‐nm shifts of λ_max_, respectively (Hiramatsu, Radlwimmer, Yokoyama, & Kawamura, 2004; Yokoyama & Radlwimmer, 1998; Yokoyama, Yang, & Starmer, 2008).

We targeted exon 3 and exon 5 for PCR and sequencing analyses. The PCR primers were set in the conserved region of the exons between the *MWS* and the *LWS* opsin genes of howler monkeys (GenBank accession numbers AB809459 and AB809460: Matsushita et al., 2014): exon 3, GGGATCACAGGTCTCTGGTC and CCTGCTCCAACCAAAGATGGG, exon 5, GCAAAGCAGCAGAAAGAGTC and CTGCCGGTTCATAAAGACATAGAT. PCR was carried out at 94°C for 5 min followed by 40 cycles at 94°C 30 s, 62°C 30 s, and at 72°C for 1 min. Pure water was used as the template for the negative control in every reaction. We directly sequenced both strands of the amplified DNA fragments using an Applied Biosystems model 3130 automatic sequencer with Big Dye Terminator v3.1 Cycle Sequencing kit and the PCR primers.

To identify hybrids, we examined the height ratio of double peaks at the three spectral‐tuning sites in the chromatogram for direct sequencing of the PCR‐amplified DNA segment. If the ratio differs strongly between the sites, the individual was expected to have a hybrid opsin gene. We confirmed hybrids by cloning and sequencing the PCR‐amplified DNA segments and used pGEM‐T vectors (Promega, Madison, WI) for DNA cloning.

### 2.4 Behavioral data collection

We collected behavioral data from one social group of mantled howlers in SSR from December through May during 1996–1997 (two adult males, seven adult females, and two juveniles) and the same social group in 1997–1998 (one adult male, four adult females, and one subadult), a time range that spanned the dry season and early rainy season in each year. Feeding records were collected using 2‐min point time samples in which all feeding animals and the plant part were identified (Altmann, 1974). All plant species consumed were recorded to the level of species, except for fig trees (genus *Ficus*). At the time that the behavioral data were collected, observers could not reliably distinguish among the *Ficus* species, although more recently these distinctions have been possible. During behavioral collection, individual monkeys were recognized based on a combination of size, pelage, and other natural markings. Some individuals also wore tagged collars (Welker, 2004).

### 2.5 Botanical collections and colorimetry

Botanical samples of dietary plants were collected from the howler monkey habitats in SSR for colorimetry in the early rainy season of 2012 (June–July). Although the botanical sampling did not overlap with the period of behavioral data collection, the diet of howler monkeys at this site has remained broadly consistent across the temporal range of this study (1997–2012; Barbara Welker unpublished data, Eugenia Zandona, unpublished data) and dietary species in 1996 through 1998 were still consumed in 2012. Leaf phenology of many species is seasonal in SSR, limiting the number of plant species from which we could collect both mature and immature leaves.

We collected spectral reflectance data for young leaves (target) and mature leaves (background) of seven dietary species at our site: *Albizia adinocephala, Albizia saman (Samanea saman), Astronium graveolens, Bursera simarouba*,* Chomelia spinosa*,* Exostema mexicana*, and *Ficus bullenoi* (Table 1), along with an additional plant species*, Gliricidia sepium*, that is frequently eaten by *A. palliata* in Mexico (Muñoz, Estrada, Naranjo, & Ochoa, 2006) and that we suspect is also eaten at our site during months outside of our observation period. Our choice of plant species was guided by the simultaneous presence of both young and mature leaves during our collection period. For both young and mature leaves, we measured the upper and lower leaf surfaces with a JAZ spectrometer (Ocean Optics Inc., Dunedin, FL). We collected five readings per surface per leaf and measured five leaves per species from a minimum of two individual trees.

**Table 1:** Percentage of foraging time, by plant part, for species consumed by mantled howler monkeys (*Alouatta palliata*) in the dry season and early wet season (December–May) in Sector Santa Rosa, ÁCG, Costa Rica

Species	Fruit %			Flowers %			Young leaves %			Mature leaves %			Total young leaf foraging (mean %)
	1997	1998	Mean	1997	1998	Mean	1997	1998	Mean	1997	1998	Mean	
***Albizia adinocephala***	0.1	0.3	0.2	0	0.4	0.2	1.9	2.1	2	0.7	0	0.35	7.6%
***Albizia (Samanea) saman***	0.3	0.8	0.55	3.5	8.6	6.05	1.2	2.1	1.65	0.6	0.5	0.55	6.2%
*Andira inermis*							0.5	0.6	0.55				2.1%
***Astronium graveolens***				0	0.1	0.05	0.8	0.7	0.75	0.8	1.3	1.05	2.8%
*Bombacopsis quinatum*				0.1	1.6	0.85							0.0%
*Brosimum aliczstrum*							<0.1	0	0	1	4.4	2.7	<0.1%
***Bursera simaruba***	0.2	2.1	1.15	2.7	7.6	5.15	3.7	0.2	1.95	0	0.2	0.1	7.4%
*Cecropia peltata*	0.1	0	0.05				0	0.1	0.05	0	<0.1	0	0.2%
***Chomelia spinosa***							0.2	0	0.1	0.2	0.3	0.25	0.4%
*Chlorophora tinctoria*							0.2	0.3	0.25	<0.1	0	0	0.9%
*Cordia alliodora*				<0.1	0	0	<0.1	0	0	0.2	0	0.1	<0.1%
*Cochlospermum vitifolium*				0	0.2	0.1							0.0%
*Enterolobium cyclocarpum*	0.1	0.2	0.15	<0.1	<0.1	0	0.1	0.1	0.1				0.4%
***Exostema mexicana***							0.1	0.6	0.35	0.1	0.3	0.2	1.3%
***Ficus*** **spp.** a	14.5	10.1	12.3				10.8	11.5	11.15	<0.1	0	0	42.2%
***Gliricidia sepium***				<0.1	0.1	0.05							0.0%
*Guazuma ulmifolia*				0.1	0	0.05	<0.1	0	0				<0.1%
*Hymenaea courbaril*							0.1	1.5	0.8				3.0%
*Inga vera*				4.8	5.1	4.95	<0.1	0.2	0.1	<0.1	0	0	0.4%
*Karwinska calderoni*	0	0.1	0.05										0.0%
*Licania arborea*	1.7	0	0.85				0.9	0	0.45				1.7%
*Lonchocarpus costaricensis*	<0.1	0	<0.05	10.4	2.9	6.65	0.1	0	0.05				0.2%
*Luehea speciosa*				0.9	<0.1	0.45	0.2	0.5	0.35				1.3%
*Lydista divaricatum*							1.7	0.6	1.15				4.3%
*Machaerium biovulatum*							1.1	1.1	1.1	0.1	0.3	0.2	4.2%
*Mastichodendron capiri*	2.1	0	1.05	1.6	1.6	1.6	<0.1	0	0	0.1	0	0.05	<0.1%
*Manikara chicle*	4.2	1	2.6	0.8	0	0.4	0	0.2	0.1				0.4%
*Pseudobombax septinatum*				0.1	0	0.05							0.0%
*Sapium glandulosum*							<0.1	0	0				<0.1%
*Sciadodendron excelsum*	<0.1	<0.1	<0.1				0.1	0	0.05				0.2%
*Simarouba glauca*	7.6	8.5	8.05	<0.1	0	0							0.0%
*Spondies mombin*	<0.1	0	<0.05	0	<0.1	0	0.2	0.2	0.2				0.8%
*Spondias purpurea*	1.3	0.8	1.05										0.0%
*Tabebuia ochracea*	<0.1	0	<0.05	3.6	0.1	1.85	<0.1	0	0				<0.1%
*Tabebuia rosea*	<0.1	<0.1	<0.1	0.1	0.1	0.1							0.0%
*Vines (unknown spp.)*	<0.1	0.1	0.1	0.2	0.5	0.35	5.3	1.1	3.2	1.2	1.7	1.45	12.1%
*Ximenia americana*	0	0.7	0.35										0.0%

Young leaves were consumed for species in gray shading. Plant species subjected to colorimetry are noted in bold.

a At the time of behavioral collection, *Ficus* trees could not be identified to the species level. Since 2007, different species of Ficus have been identified in SSR and howler monkeys have been seen consuming *Ficus bullenoi,* which is subjected to colorimetry in this study. However, the proportion of this diet comprised by the different species of Ficus is presently unknown.

### 2.6 Chromaticity plots and JND models of conspicuity

Chromaticity plots and calculations of just‐noticeable differences (JNDs) are widely used in the study of primate visual ecology (Higham et al., 2010; Hiramatsu et al., 2009; Melin, Hiramatsu et al., 2014; Osorio, Smith, Vorobyev, Buchanan‐Smith, & Ryan, 2004; Osorio & Vorobyev, 1996; Pessoa et al., 2014; Valenta et al., 2015). We calculated the quantum catch of photons by the LWS, MWS, and SWS cones following established methods, reported in detail elsewhere (Hiramatsu et al., 2008; Matsumoto et al., 2014). The peak sensitivity values (λ_max_) for MWS (532 nm), LWS (564 nm), and an M‐L hybrid (547 nm) photopigment have been previously measured for howler opsins (Matsushita et al., 2014). We used a SWS photopigment λ_max_ value of 435 nm, which is intermediate to values estimated for *Ateles* (432 nm) and *Lagothrix* (437 nm) species, the most closely related taxa measured to date (Jacobs & Deegan, 2001). To assess the chromatic conspicuity of young leaves against a mature leaf background, we calculated chromatic distance between target and background along the red‐green (L/(M + L)) and blue‐yellow (S/(M + L)) chromatic axes, and visualized the values in 2‐dimensional chromaticity plots. To account for achromatic aspects of color, we calculated and plotted luminance (log (M + L)) for each plant species.

We conducted a JND analysis to predict whether targets exceeded a threshold in their color difference from background leaves. The JND calculation models chromatic differences in the color space of different phenotypes, and accounts for sources of neural noise. We followed the bright‐light version of this model (Osorio et al., 2004), as our observations were conducted strictly during diurnal periods. We interpret the color of a target to be visible if it differs from the background by 1 JND. Furthermore, a difference of 1 JND or greater between any two phenotypes suggests a significant difference that could translate into a sensory advantage. The value of 1 JND is based on data from humans in laboratory conditions (Sperling & Harwerth, 1971), and an assumption of our study is that this value is also meaningful for other primates with highly acute color vision, such as howler monkeys (Muniz, de Athaide, Gomes, Finlay, & Silveira, 2014; Salazar, Dominy, & Laska, 2015). The illuminance spectrum for all analyses was measured under “forest shade” at our study site using a USB 2000 spectrophotometer with a cosine corrector (Ocean Optics Inc., Dunedin, FL) (Hiramatsu et al., 2008). All analyses were carried out in MATLAB R2014a. To correct for spectrometer drift in baseline (zero reference) during measurements, all spectra were normalized prior to analysis through application of a custom spline function setting the baseline value to zero. We performed separate JND calculations for standard trichromats *T*_Stand_, anomalous trichromats *T*_Anom_, and also for (hypothetical) dichromats—that is, the phenotype resulting if a monkey possessed only an LWS opsin (*D*_L_), or an MWS opsin (*D*_M_).

### 2.7 Seasonality in young leaf abundance

To explore the seasonal patterns of young leaf flushing among species in the deciduous dry forests of the Área de Conservación Guanacaste, we queried long‐term phenological records that have been collected in Sector Santa Rosa since 2007 on 41 tree species. Although these phenological records do not specifically monitor howler monkey food species, many tree species monitored are consumed by howlers (Table 1). The phenology records consist of monthly estimates of leaf coverage and maturity on a median of eight individual trees of each species in each month. We calculated monthly availability indices of new leaves and of mature leaves from each phenology record following Campos et al., (2014). In brief, the availability index calculates the mean proportion of the tree canopy covered by the phenophase of interest for each species, each month. To better capture the broad seasonal pattern of leaf availability, we applied a loess smoother to the raw monthly availability indices for each species to devalue the influence of individual trees that were outliers and to allow the availability scores in adjacent months to moderate influence on each other. We calculated the peak in leaf availability for each species by treating the 12 months of the year as circular and then calculating the mean resultant vector weighted by the smoothed availability scores. The direction of the mean vector indicates the timing of the peak in availability, and the magnitude of the vector combines information about the degree of synchrony and the level of availability. Low‐magnitude vectors reflect low overall availability and/or asynchronous availability, whereas the highest magnitudes indicate large and highly synchronous availability scores.

## 3 Results

### 3.1 Dietary composition

Howler monkeys consumed the young leaves of 28 plant species, with strong biases toward a subset of the species (Table 1, gray shading). The species represented in our collection for colorimetry made up 67% of total young leaves consumption records (Table 1 *in bold)*. Mature leaves of a subset of total dietary species were also consumed, but typically when young leaves were absent. Howlers consumed the fruits or flowers of an additional 10 plant species during the period of our behavioral study (Table 1).

### 3.2 Opsin genotypes

**Figure 2:**
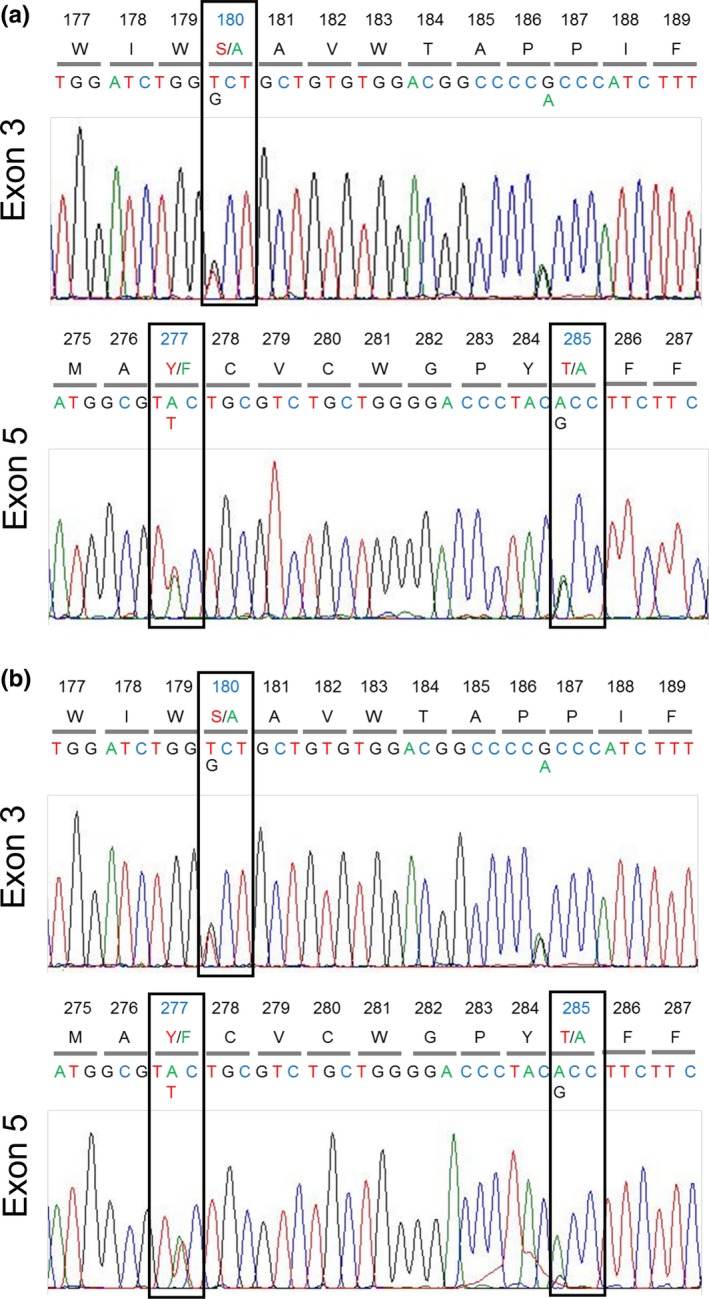
Exemplary chromatograms of direct PCR‐sequencing for partial exon 3 and exon 5 of *M/LWS* opsin genes from (a) a female howler monkey with one standard *LWS* and one standard *MWS* opsin genes on each X chromosome and (b) a female howler with one X chromosome carrying a standard *LWS* and a standard *MWS* opsin genes and the other X chromosome carrying a standard *LWS* opsin gene and an M‐L hybrid opsin gene. Amino acid site number is indicated in the top row. Amino acid abbreviations are given in the second row. The nucleotide reads are indicated in the third row. Letters and chromatograms for adenine, guanine, cytosine, and thymine are colored with green, black, blue, and red, respectively. The three spectral tuning amino acid site numbers, 180, 277, and 285 are highlighted in blue and enclosed in boxes. The L‐type and M‐type amino acids at the three sites are colored with red and green, respectively. Amino acid heterozygosity is apparent at the three sites by the double peaks. Note that double peaks are approximately the same height except at site 285 of the normal plus hybrid opsin gene (b)

We successfully sequenced the opsin genes of five male and three female howlers (eleven X‐chromosomes in total) spanning four social groups (Table 2). Four of the samples from the 12 individuals containing >200 pg μl^−1^ DNA did not amplify well, and we could not generate clear sequences. Opsin gene sequences for two females have previously been published (Matsushita et al., 2014). We examined the sequence chromatograms to detect opsin variation. The majority of individuals showed the same relative height of double peaks in the chromatogram at the three spectral‐tuning sites (180, 277, and 285) as exemplified in Figure 2a. We detected standard L‐type and standard M‐type exon 5 sequences but did not detect any hybrid exon 5 sequences in the DNA clones from these samples. Thus, we rule out the possibility of coexistence of reciprocal types of hybrid exon 5 in these individuals. Coexistence of reciprocal types of recombinants between exons 3 and 5 cannot be ruled out. However, we assumed these individuals to possess standard M‐ and standard L‐type opsin genes to make a conservative estimate of incidence of hybrid opsins.

**Table 2:** Details of opsin sampling and genotyping for four social groups of mantled howler monkeys (*Alouatta palliata*) in Sector Santa Rosa, ÁCG, Costa Rica. Samples in bold (CG‐17 and BH‐10) have L/M hybrid opsins

Group	Sex	Number of individuals genotyped	Individual ID	Number of X‐ chromosomes	Number of L/M hybrid opsins	Frequency of hybrid opsin genes per X‐chromosome (%)
CG	Male	1	CG‐19	1	0	0
	Female	2	CG‐12, **CG‐17**	4	1	25
BH	Male	1	BH‐24	1	0	0
	Female	1	**BH‐10**	2	1	50
SE	Male	1	SE‐30	1	0	0
CJ	Male	2	CJ‐01, CJ‐04	2	0	0
Total		8		11	2	18.2

In addition to the one female howler (CG‐17) possessing the M‐L hybrid exon 5 (M‐type amino acid at 277 and L‐type amino acid at 285) reported in Matsushita et al. (2014), one female from BH group (BH‐10) possessed a hybrid opsin. The chromatogram showed approximately the same height of the double peaks at sites 180 and 277, but approximately 3:1 height ratio of double peaks at site 285 [3 A to 1 G, resulting in 3 Thr (L‐type) to 1 Ala (M‐type)] (Figure 2a). We confirmed BH‐10 possesses the same M‐L type hybrid exon 5 sequence as CG‐17, as well as the standard L‐type and standard M‐type sequences, through DNA cloning. We conservatively conclude that one of the two X chromosomes of these females carries one normal L and one normal M opsin gene. If we also make another conservative assumption that the second X chromosome carries one normal L opsin gene, then the other opsin gene on this chromosome has to be a hybrid opsin gene in which the sites 180 and 277 are occupied with M‐type amino acids and the site 285 is occupied with L‐type amino acid to account for the 3 L to 1 M ratio at site 285. Thus, this hybrid gene is the “Apa_M_A285T” variant, for which the λ_max_ is known to be 547 nm (Matsushita et al., 2014). The incidence of the hybrid *M/LWS* opsin in SSR is two of 11 X‐chromosomes (18%). Including results of *A. palliata* samples (4 X‐chromosomes) from Nicaraguan individuals that have been previously reported, the incidence of the hybrid *M/LWS* opsin genes in this species is 13.3%, slightly increased from the prior estimate of 12.5% (Matsushita et al., 2014).

### 3.3 Chromatic conspicuity of young leaves

**Figure 3:**
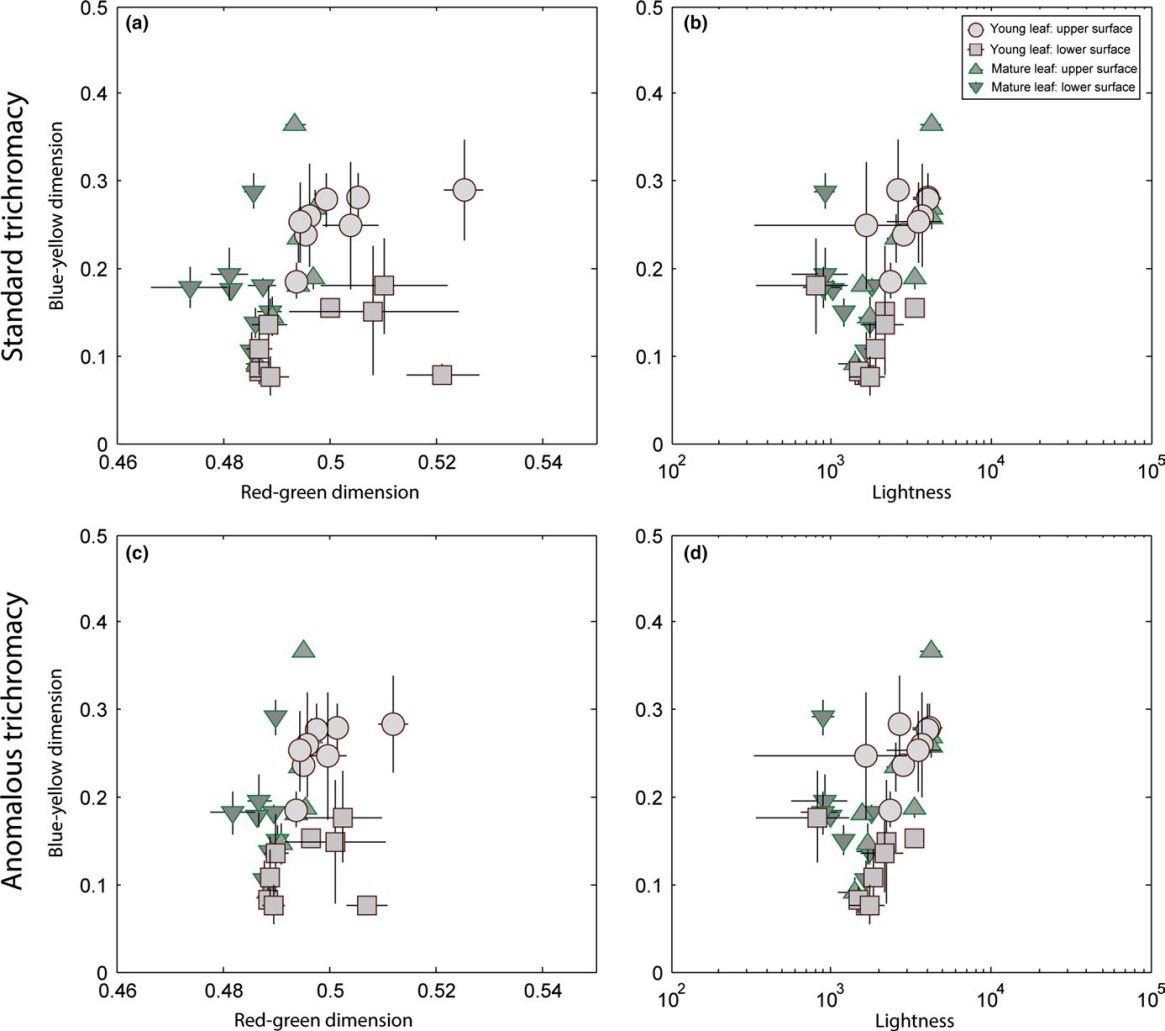
Chromaticity and luminance plots of eight dietary species. Chromaticity values show the color distance for (a) the standard trichromat phenotype and (b) the anomalous color vision phenotype along the red‐green color dimension (*x*‐axis; L/(L + M)) and along the blue‐yellow dimension (*y*‐axis; S/(L + M)). Greater values along the *x*‐axis indicate redder coloration, and greater values along the *y*‐axis indicate bluer coloration. Luminance values (*x*‐axis; log(L + M)) are plotted against the blue‐yellow chromatic axis (*y*‐axis) for (c) the standard trichromat phenotype and (d) the anomalous color vision phenotype. Lighter gray data points plot young leaves (circles—upper surface, squares—lower surface), and darker gray triangles plot mature leaves. Upper mature leaves are marked by upward facing triangles, lower mature leaves are marked by downward facing triangles

Young leaves of several species appeared reddish to human eyes and reflected a greater percentage of longer wavelengths, relative to mature leaves (Figure 1b, inset). Chromaticity diagrams plot the mean and standard deviation of the chromaticity values of each plant part (young leaf, mature leaf) and surface (upper, lower) for a standard howler monkey trichromatic phenotype as well as the anomalous trichromatic phenotype (Figure 3). The young leaves of three species—*Albizia (Samanea) saman*,* Astronium graveolens, and Ficus bullenoi*—were chromatically well separated from mature leaves along the red‐green axis (Figure 3a,c). One species was on the border of the red‐green color space occupied by mature leaves (*Gliricidia sepuium*), and the remaining three species fell within the range of mature leaf chromaticity. No species had young leaves that were well separated from mature leaf values along the blue‐yellow chromatic axis or along the luminance axis (Figure 3b,d). Similar patterns were observed for both upper and lower young leaf surfaces. Patterns for the anomalous trichromat (Figure 3c,d) were broadly similar to the standard trichromatic phenotype (Figure 3a,b), although with slightly less chromatic separation along the red‐green axis.

**Figure 4:**
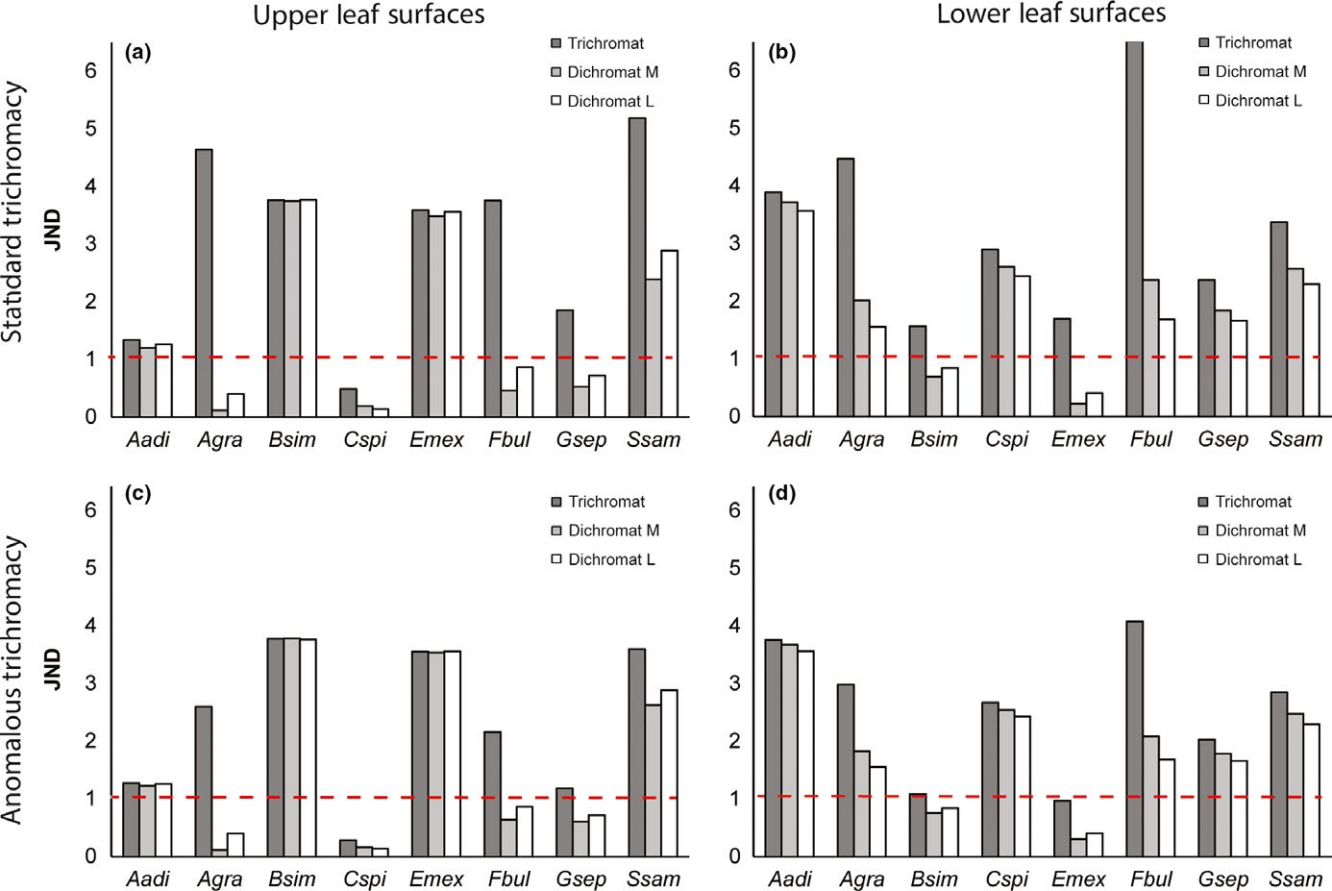
JND analysis histogram for young leaves observed against mature leaves: (a,c) upper leaf surfaces; (b,d) lower leaf surfaces. Histogram shows JND analysis in dark gray for standard trichromats (a,b) (T_LM_) or anomalous trichromats (c,d), and in moderate gray for Dichromat M (D_M_) and white for Dichromat L (D_L_). The young leaves of species with less than one JND value (represented by a dashed line in both figures) are not predicted to be discernable to observers. A difference of more than one JND value between phenotypes signifies a predicted perceptual advantage. *Albizia adinocephala* (Aade)*, Albizia saman* (*Samanea saman*; Ssam)*, Astronium graveolens* (Agra)*, Bursera simarouba* (Bsim), *Chomelia spinose* (Cspi), *Exostema Mexicana* (Emex), and *Ficus bullenoi* (Fbul), *Gliricidia sepium* (Gsep) (Table 1)

Our analysis indicates that chromatic differences between young and mature leaves should be detectable (JND > 1) to trichromatic howler monkeys for all eight plant species measured through colorimetry. However, for one species, *Chomelia spinosa*, only the lower surface of the leaves had a JND value >1 for trichromats (Figure 4). JND analysis for dichromats (D_M_ or D_L_) yielded values >1 for the upper leaf surfaces of four species: *Albizia adinocephala, Albizia saman* (*Samanea saman*), *Bursera simaruba, Exostema mexicana,* and for the lower surfaces of six species (all but *Bursera simaruba* and *Exostema mexicana*). The JND values for dichromats were at the boundary of the 1 JND threshold in many cases.

JND comparisons can also be used to assess how much more conspicuous a color difference is to one phenotype versus another. Overwhelmingly, the JND values for dichromats were below the respective values for trichromats (Figure 4). In particular, trichromats should have a perception advantage (>1 JND) when searching for *Astronium gaveolens, Ficus bullenoi*,* Albizia saman* (*Samanea saman*), and *Gliricidia sepium*. If leaf orientation on the tree is variable, such that lower sides are also seen frequently, trichromats would also have an advantage for *Exostema mexicana* and possibly *Bursera simaruba*. The dichromatic phenotypes did not differ from each other by more than one JND for any of the plant species in our color discrimination models. This suggests that neither of the hypothetical dichromatic phenotypes would be more advantageous than the other for detecting young leaves against a mature leaf background. The values of the anomalous trichromat with the hybrid M‐L opsin were between the values of the standard trichromat and those of the dichromatic phenotypes, but more closely resembled the trichromat detection performance (Figure 4). Anomalous trichromats would not likely have an advantage over dichromats for *Exostema mexicana* or *Bursera simaruba*.

### 3.4 Seasonality in young leaf abundance

**Figure 5:**
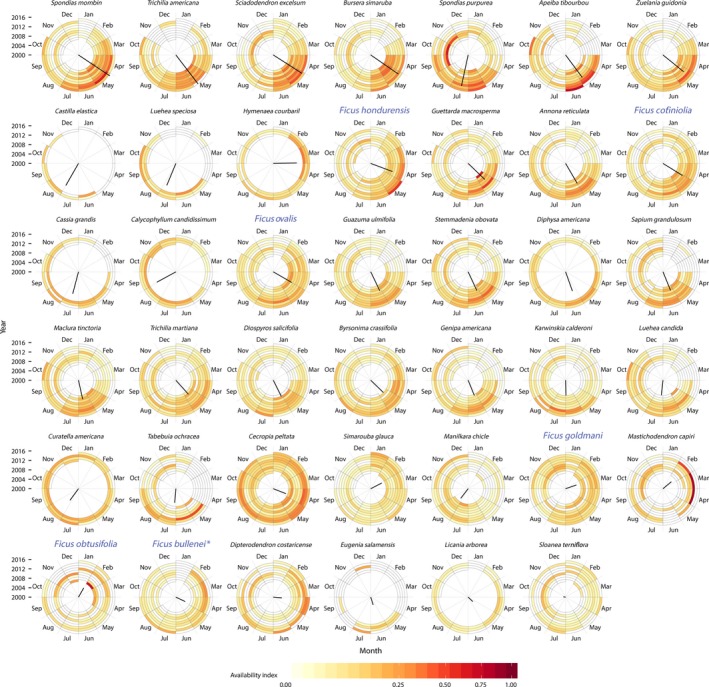
Circular heat maps of young leaf availability for 41 tree species in Sector Santa Rosa ordered from most to least synchronous in annual young leaf flush. The colored tiles show loess‐smoothed estimates of young‐leaf availability for each species, based on an availability index calculated for each individual tree of that species that was scored in that particular month and year (Campos et al., 2014). The availability index is an estimate of the proportion of the tree's canopy that is covered by new leaves. Fig trees (*Ficus* species, blue enlarged typeface) are among the most asynchronous and are often flush in young leaves when other species are not

**Figure 6:**
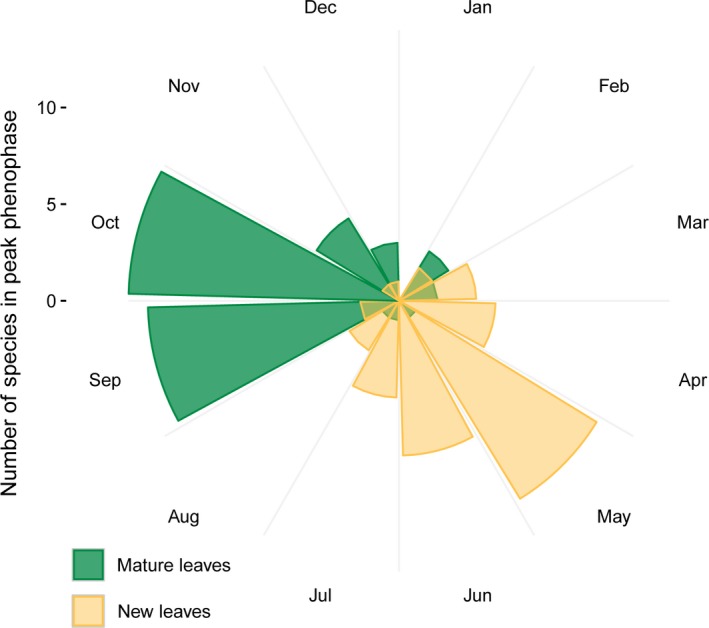
Circular histograms showing the number of monitored plant species that are in peak phenophase during each month for mature leaves (green) and young leaves (orange). The month of peak phenophase for each species is based on monthly phenological records collected over a 9‐year period. The peak in young leaf availability coincides with the dry‐to‐wet season transition in May, while the peak in mature leaf availability coincides with the peak of the wet season in September

A majority of plant species, including *Bursera simaruba,* an important species in the howler diet, shows strongly seasonal new leaf flush, while other species including several *Ficus* species and *Cecropia peltata* (another member of the Moracea family) in SSR have an asynchronous and aseasonal pattern (Figure 5). These species are often flush with new leaves during periods of the annual cycle with low availability of young leaf resources (Figure 6).

## 4 Discussion

### 4.1 Advantage to trichromatic primates in foraging for young leaves

Our study of the foraging ecology, opsin genes, and models of color vision performance of mantled howlers leads us to three primary findings: (1) many plant species important to the howler diet possess young leaves that are reddish and more chromatically distinct from mature green leaves to trichromatic phenotypes than to dichromatic phenotypes; (2) all howlers examined possessed distinct M‐ and L‐type opsin genes, providing the genetic basis for trichromatic vision. We reinforce a previous report (Matsushita et al., 2014) that some howlers possess hybrid opsins, leading to anomalous trichromacy if possessed by males, and find that opsin hybrids are relatively common (13% of X‐chromosomes); and (3) standard trichromacy was modeled to be more advantageous than anomalous trichromacy, but the latter still performed well in JND analyses, and considerably better than modeled dichromats.

The genus *Alouatta* is unique among New World primates in that all males and females are capable of trichromatic vision (routine trichromacy), while all other diurnal platyrrhine species examined are comprised of mixed populations of dichromats and trichromats (polymorphic trichromacy). Howler monkeys are also acknowledged as being the most folivorous genus in the Neotropics (Chapman, 1988; Milton, 1980; Rosenberger et al., 2011), and our results lend support for the folivory hypothesis of routine trichromacy (Dominy & Lucas, 2001). Trichromatic vision is suited for identifying many young leaves from a background of mature leaves, especially when viewing upper leaf surfaces. That howlers may rely more heavily on leaves when fruit is scarce (Behie & Pavelka, 2015) highlights the potential role of young leaves as a high‐quality fall‐back food (Lucas et al., 2003; Marshall, Boyko, Feilen, Boyko, & Leighton, 2009), which aligns with another central pillar of the folivory hypothesis. While we did not model fruit perception in this study, numerous other studies have demonstrated that howlers eat colorful fruits and have identified the potential for trichromat advantage in fruit perception (e.g., Martins, 2007; Melin, Hiramatsu et al., 2014; Osorio et al., 2004; Regan et al., 1998); thus, benefits may extend to this resource as well. Limitations of our study include the restriction of behavioral observation to 6 months of the year and the temporal incongruity of behavioral observations with opsin genotyping, which prohibited linking foraging performance with color vision phenotype. In the future, behavioral, genetic, and colorimetric studies should happen simultaneously and span the full annual cycle. Analysis of the nutritional and mechanical properties of immature versus mature leaves would also further our understanding of the importance of color as a foraging cue to howlers.

### 4.2 *Ficus* as a potential keystone resource for trichromatic folivores

New leaves are a nutrient‐rich (Milton, 1979) but relatively rare and fleeting resource (Welker, 2004), and there is likely strong selective pressure to capitalize on them when they are available. The ubiquitous and strong trichromatic advantage predicted for detecting the young leaves of *Ficus bullenoi* is particularly notable (Figure 4). Young *Ficus* leaves are reported to be an important young leaf food for howlers (Serio‐Silva, Rico‐Gray, Hernández‐Salazar, & Espinosa‐Gómez, 2002), and they can account for nearly half of the folivorous components of the howler monkey diet (Cristóbal‐Azkarate & Arroyo‐Rodríguez, 2007; Milton, 1980). Importantly, and unlike many other sympatric taxa, *Ficus* species flush their leaves asynchronously and aseasonally, and they may provide young, nutritious leaves at times when other species may only possess uniformly mature phenophase, or have lost their leaves completely (Milton, 1991; Pereira, Rodrigues, & de Oliveira Menezes, 2006). Here, we show that few plant species produce a large amount of new leaves during the late wet season to mid dry season (roughly August to March). This period overlaps with the typical period of greatest fruit scarcity in SSR (Melin, Young, et al., 2014). During this time, the small set of trees species that produce new leaves, including *Ficus* species, may be particularly important to howlers. A keystone role of *Ficus* spp. in tropical ecosystems has been well documented, although these studies tend to focus on the importance of the fig “fruits” (Dominy et al., 2003; Janzen, 1979; Parr et al., 2011; Valenta & Melin, 2012; Yang et al., 2002); our data suggest a critical role of *Ficus* and other plant taxa that produce leaves aseasonally, or in the late wet/early dry period as a key resource to folivorous animals. Future study will reveal the importance of *Ficus bullenoi* to howler monkeys relative to other *Ficus* species, and the extent to which young reddish leaves are characteristic of this genus. In addition, greater knowledge of howler diets and food abundance in SSR and other habitats will better allow us to understand how these monkeys survive lean periods and reveal selection pressures shaping their dietary and sensory adaptations.

### 4.3 Implications for the evolution of routine trichromacy

That howler monkeys are the only neotropical monkey with routine trichromacy has puzzled sensory ecologists since its discovery in 1996 (Jacobs et al., 1996). Competing hypotheses include the possibilities that: (1) other neotropical primate taxa have not experienced the necessary genetic event, that is, juxtaposition of the two divergent *M/LWS* opsin alleles, leading to trichromacy in male carriers as well as females; (2) these unequal crossover events have occurred in other lineages, but that insufficient advantage in combination with other stochastic events resulted in their loss from the population; (3) routine trichromacy was fixed in the early population of howler monkeys solely by random genetic drift, or (4) trichromacy was fixed in howler monkeys due to increased natural selection favoring routine trichromacy. Although we cannot conclusively reject hypotheses 1–3 given the design and scope of the present study, our results support the fourth hypothesis. Selection for routine trichromacy may be higher in howler monkeys than in other New World monkeys for at least two reasons. First, they experience strong benefits from red‐green color vision for folivory and, although we did not assess fruit foraging in this study, trichromacy is likely also beneficial for the frugivorous aspects of their diet (e.g., Bompas, Kendall, & Sumner, 2013; Melin et al., 2009; Mollon, 1989; Regan et al., 1998). Second, the persistence of dichromacy may not be favored in howlers. We briefly consider this latter point below and offer it as a hypothesis for future testing.

Well‐camouflaged invertebrate prey, and perhaps other cryptic foods including immature seeds from greenish, unripe fruit, provide essential protein in the diets of many New World primates, for example, capuchins, tamarins, squirrel monkeys, and muriquis, but these foods do not form a large part of the howler diet (Martins, 2007; Rothman, Raubenheimer, Bryer, Takahashi, & Gilbert, 2014). Dichromatic advantage for capturing cryptic invertebrates has been found and is argued to maintain the presence of dichromats in other platyrrhine species via balancing selection (Hiwatashi et al., 2010; Kawamura et al., 2012; Melin, Fedigan, Young, & Kawamura, 2010; Melin et al., 2007; Smith et al., 2012). Because trichromatic howler monkeys primarily obtain their protein from leaves (Amato & Garber, 2014), they may not experience a disadvantage of having a population solely comprised of trichromats, who should be able to locate both fruits and leaves. Even for plant species in which young leaves are greenish, the smaller leaf size and/or distinctive shape would be a visual cue accessible to both dichromats and trichromats. In the absence of strong situational advantage to dichromatic group members, it is therefore possible that routine trichromacy was more likely to become fixed in howler monkeys. A similar scenario may also have contributed to the fixation of routine trichromacy in Old World monkeys, if leaves—more so than invertebrates—were a particularly important food source during periods of food dearth. In future studies, this could be tested by carefully examining cryptic food consumption by howlers and other primates, in combination with evaluating the strength of purifying selection to maintain intact *LWS* and *MWS* opsin genes against gene conversion (e.g., Hiwatashi et al., 2011). More directly, measurement of intraspecific diversity and interspecific divergence of the *M/LW*S opsin gene region in genomewide scans could detect signatures of ancient selection (skewed to short coalescent time and small apparent population size in the *M/LWS* opsin gene region).

The incidence of hybrid opsin genes is higher in howler monkeys than in Old World primates examined thus far. If evidence of stronger purifying selection in Old World monkeys is also found, this may reflect a more important role of reddish coloration in the visual ecology of catarrhine primates. A likely candidate for this pressure is widespread use of reddish colors in sociosexual communication among this group. This novel color role appears to be an important and widespread adaptation in catarrhine evolution (Changizi, Zhang, & Shimojo, 2006; Dubuc, Allen, Maestripieri, & Higham, 2014; Higham et al., 2010; Setchell, Wickings, & Knapp, 2006), which arose following the origin of trichromacy in a foraging context (Fernandez & Morris, 2007).

## 5 Summary

Like their Old World relatives, we find that plants consumed by primates in the Neotropics often produce young leaves that are more reddish than mature leaves. Our models of howler monkey color vision indicate that trichromacy would be adaptive to these primates for locating this nutritious resource. We find a relatively high incidence of hybrid *M/LWS* opsin genes in *Alouatta palliata*, the mantled howler monkey; the resulting anomalous phenotype still performs relatively well in models of food detection, and this phenotype may not experience strong negative selection. We suggest that routine trichromacy in howlers may have been favored in the absence of balancing selection maintaining dichromatic individuals, which appears to be happening in other species of neotropical monkeys. Further, comparative studies of the opsin genes, diet, and social communication mechanisms of platyrrhine and catarrhine species will continue to elucidate the selection pressures acting on primate color vision.

## Conflict of Interest

The authors have no competing interests to declare.

## References

[ece32716-bib-0001] Altmann, J. (1974). Observational study of behavior: Sampling methods. Behaviour, 49, 227–267. 4597405 10.1163/156853974x00534

[ece32716-bib-0002] Amato, K. R. , & Garber, P. A. (2014). Nutrition and foraging strategies of the black howler monkey (*Alouatta pigra*) in Palenque National Park, Mexico. American Journal of Primatology, 76, 774–787. 24763976 10.1002/ajp.22268

[ece32716-bib-0003] Behie, A. M. , & Pavelka, M. S. M. (2015). Fruit as a key factor in howler monkey population density: Conservation implications In Kowalewski M. M., Garber P. A., Cortés‐Ortiz L., Urbani B., & Youlatos D. (Eds.), Howler monkeys (pp. 357–382). New York: Springer.

[ece32716-bib-0004] Bennett, A. T. D. , & Thery, M. (2007). Avian color vision and coloration: Multidisciplinary evolutionary biology. The American Naturalist, 169, S1–S6.

[ece32716-bib-0005] Bompas, A. , Kendall, G. , & Sumner, P. (2013). Spotting fruit versus picking fruit as the selective advantage of human colour vision. i‐Perception, 4, 84–94. 23755352 10.1068/i0564 PMC3677335

[ece32716-bib-0006] Bowmaker, J. K. (1998). Evolution of colour vision in vertebrates. Eye, 12, 541–547. 9775215 10.1038/eye.1998.143

[ece32716-bib-0007] Campbell C. J., Fuentes A., MacKinnon K., Bearder S., & Stumpf R. (Eds.) (2011). Primates in perspective. Second. Oxford University Press: New York.

[ece32716-bib-0008] Campos, F. A. , Bergstrom, M. L. , Childers, A. , Hogan, J. D. , Jack, K. M. , Melin, A. D. , … Fedigan, L. M. (2014). Drivers of home range characteristics across spatiotemporal scales in a neotropical primate, *Cebus capucinus* . Animal Behaviour, 91, 93–109.

[ece32716-bib-0009] Campos, F. A. , & Fedigan, L. M. (2009). Behavioral adaptations to heat stress and water scarcity in white‐faced capuchins (*Cebus capucinus*) in Santa Rosa National Park, Costa Rica. American Journal of Physical Anthropology, 138, 101–111. 18711741 10.1002/ajpa.20908

[ece32716-bib-0010] Campos, F. A. , Jack, K. M. , & Fedigan, L. M. (2015). Climate oscillations and conservation measures regulate white‐faced capuchin population growth and demography in a regenerating tropical dry forest in Costa Rica. Biological Conservation, 186, 204–213.

[ece32716-bib-0011] Changizi, M. A. , Zhang, Q. , & Shimojo, S. (2006). Bare skin, blood and the evolution of primate colour vision. Biology Letters, 2, 217–221. 17148366 10.1098/rsbl.2006.0440 PMC1618887

[ece32716-bib-0012] Chapman, C. (1988). Patterns of foraging and range use by three species of neotropical primates. Primates, 29, 177–194.

[ece32716-bib-0013] Cristóbal‐Azkarate, J. , & Arroyo‐Rodríguez, V. (2007). Diet and activity pattern of howler monkeys (*Alouatta palliata*) in Los Tuxtlas, Mexico: Effects of habitat fragmentation and implications for conservation. American Journal of Primatology, 69, 1013–1029. 17330311 10.1002/ajp.20420

[ece32716-bib-0014] Dominy, N. J. , & Lucas, P. W. (2001). Ecological importance of trichromatic vision to primates. Nature, 410, 363–366. 11268211 10.1038/35066567

[ece32716-bib-0015] Dominy, N. J. , & Lucas, P. W. (2004). Significance of color, calories, and climate to the visual ecology of catarrhines. American Journal of Primatology, 62, 189–207. 15027092 10.1002/ajp.20015

[ece32716-bib-0016] Dominy, N. J. , Svenning, J. C. , & Li, W. H. (2003). Historical contingency in the evolution of primate color vision. Journal of Human Evolution, 44, 25–45. 12604302 10.1016/s0047-2484(02)00167-7

[ece32716-bib-0017] Dubuc, C. , Allen, W. L. , Maestripieri, D. , & Higham, J. P. (2014). Is male rhesus macaque red color ornamentation attractive to females? Behavioral Ecology and Sociobiology, 68, 1215–1224. 25246728 10.1007/s00265-014-1732-9 PMC4167843

[ece32716-bib-0018] Dulai, K. S. , von Dornum, M. , Mollon, J. D. , & Hunt, D. M. (1999). The evolution of trichromatic color vision by opsin gene duplication in New World and Old World primates. Genome Research, 9, 629–638. 10413401

[ece32716-bib-0019] Espinosa‐Gómez, F. , Gómez‐Rosales, S. , Wallis, I. R. , Canales‐Espinosa, D. , & Hernández‐Salazar, L. (2013). Digestive strategies and food choice in mantled howler monkeys *Alouatta palliata mexicana*: Bases of their dietary flexibility. Journal of Comparative Physiology B, Biochemical, Systemic, and Environmental Physiology, 183, 1089–1100. 10.1007/s00360-013-0769-9 23743799

[ece32716-bib-0020] Estrada A., Garber P. A., Pavelka M. S. M., & Luecke L. (Eds.) (2006). new perspectives in the study of Mesoamerican primates. US, Boston, MA: Springer.

[ece32716-bib-0021] Fedigan, L. M. , & Jack, K. M. (2012). Tracking neotropical monkeys in Santa Rosa: Lessons from a regenerating Costa Rican dry forest In Kappeler P. M., & Watts D. P. (Eds.), Long‐term field studies of primates (pp. 165–184). Berlin Heidelberg: Springer.

[ece32716-bib-0022] Fernandez, A. A. , & Morris, M. R. (2007). Sexual selection and trichromatic color vision in primates: Statistical support for the preexisting‐bias hypothesis. The American Naturalist, 170, 10–20. 10.1086/518566 17853988

[ece32716-bib-0023] Higham, J. P. , Brent, L. J. N. , Dubuc, C. , Accamando, A. K. , Engelhardt, A. , Gerald, M. S. , … Stevens, M. (2010). Color signal information content and the eye of the beholder: A case study in the rhesus macaque. Behavioral Ecology, 21, 739–746. 22475874 10.1093/beheco/arq047 PMC2892627

[ece32716-bib-0024] Hiramatsu, C. , Melin, A. D. , Aureli, F. , Schaffner, C. M. , Vorobyev, M. , & Kawamura, S. (2009). Interplay of olfaction and vision in fruit foraging of spider monkeys. Animal Behaviour, 77, 1421–1426.

[ece32716-bib-0025] Hiramatsu, C. , Melin, A. D. , Aureli, F. , Schaffner, C. M. , Vorobyev, M. , Matsumoto, Y. , & Kawamura, S. (2008). Importance of achromatic contrast in short‐range fruit foraging of primates. PLoS ONE, 3, e3356. 18836576 10.1371/journal.pone.0003356 PMC2559900

[ece32716-bib-0026] Hiramatsu, C. , Radlwimmer, F. B. , Yokoyama, S. , & Kawamura, S. (2004). Mutagenesis and reconstitution of middle‐to‐long‐wave‐sensitive visual pigments of New World monkeys for testing the tuning effect of residues at sites 229 and 233. Vision Research, 44, 2225–2231. 15208009 10.1016/j.visres.2004.04.008

[ece32716-bib-0027] Hiramatsu, C. , Tsutsui, T. , Matsumoto, Y. , Aureli, F. , Fedigan, L. M. , & Kawamura, S. (2005). Color‐vision polymorphism in wild capuchins (*Cebus capucinus*) and spider monkeys (*Ateles geoffroyi*) in Costa Rica. American Journal of Primatology, 67, 447–461. 16342069 10.1002/ajp.20199

[ece32716-bib-0028] Hiwatashi, T. , Mikami, A. , Katsumura, T. , Suryobroto, B. , Perwitasari‐Farajallah, D. , Malaivijitnond, S. , … Kawamura, S. (2011). Gene conversion and purifying selection shape nucleotide variation in gibbon L/M opsin genes. BMC Evolutionary Biology, 11, 312. 22017819 10.1186/1471-2148-11-312 PMC3213168

[ece32716-bib-0029] Hiwatashi, T. , Okabe, Y. , Tsutsui, T. , Hiramatsu, C. , Melin, A. D. , Oota, H. , … Kawamura, S. (2010). An explicit signature of balancing selection for color‐vision variation in new world monkeys. Molecular Biology and Evolution, 27, 453–464. 19861643 10.1093/molbev/msp262

[ece32716-bib-0031] Jacobs, G. H. (1996). Primate photopigments and primate color vision. Proceedings of the National Academy of Sciences of the United States of America, 93, 577–581. 8570598 10.1073/pnas.93.2.577 PMC40094

[ece32716-bib-0032] Jacobs, G. H. , & Deegan, J. F. (2001). Photopigments and colour vision in New World monkeys from the family Atelidae. Proceedings of the Royal Society B: Biological Sciences, 268, 695–702. 11321057 10.1098/rspb.2000.1421 PMC1088658

[ece32716-bib-0033] Jacobs, G. H. , Neitz, M. , Deegan, J. F. , & Neitz, J. (1996). Trichromatic colour vision in New World monkeys. Nature, 382, 156–158. 8700203 10.1038/382156a0

[ece32716-bib-0034] Jacobs, G. H. , & Rowe, M. P. (2004). Evolution of vertebrate colour vision. Clinical and Experimental Optometry, 87, 206–216. 15312024 10.1111/j.1444-0938.2004.tb05050.x

[ece32716-bib-0035] Janzen, D. H. (1979). How to be a Fig. Annual Review of Ecology and Systematics, 10, 13–51.

[ece32716-bib-0036] Janzen, D. H. (1988). Tropical dry forests: The most endangered major tropical ecosystem In Wilson E. O. (Ed.), Biodiversity (pp. 130–137). Washington, D.C.: National Academy of Sciences.

[ece32716-bib-0037] Johnsen, S. , Kelber, A. , Warrant, E. , Sweeney, A. M. , Widder, E. A. , Lee, R. L. , & Hernández‐Andrés, J. (2006). Crepuscular and nocturnal illumination and its effects on color perception by the nocturnal hawkmoth *Deilephila elpenor* . Journal of Experimental Biology, 209, 789–800. 16481568 10.1242/jeb.02053

[ece32716-bib-0038] Kawamura, S. , Hiramatsu, C. , Melin, A. D. , Schaffner, C. M. , Aureli, F. , & Fedigan, L. (2012). Polymorphic color vision in primates: Evolutionary considerations In Hirai H., Imai H., & Go Y. (Eds.), Post‐genome biology of primates, primatology monographs (pp. 93–120). Tokyo, Japan: Springer.

[ece32716-bib-0039] Lucas, P. W. , Dominy, N. J. , Riba‐Hernandez, P. , Stoner, K. E. , Yamashita, N. , Loría‐Calderón, E. , … Darvell, B. W. (2003). Evolution and function of routine trichromatic vision in primates. Evolution; International Journal of Organic Evolution, 57, 2636–2643. 14686538 10.1111/j.0014-3820.2003.tb01506.x

[ece32716-bib-0040] Marshall, A. J. , Boyko, C. M. , Feilen, K. L. , Boyko, R. H. , & Leighton, M. (2009). Defining fallback foods and assessing their importance in primate ecology and evolution. American Journal of Physical Anthropology, 140, 603–614. 19890868 10.1002/ajpa.21082

[ece32716-bib-0041] Martins, M. M. (2007). Fruit diet of *Alouatta guariba* and *Brachyteles arachnoides* in Southeastern Brazil: Comparison of fruit type, color, and seed size. Primates, 49, 1–8. 17578654 10.1007/s10329-007-0050-5

[ece32716-bib-0042] Matsumoto, Y. , Hiramatsu, C. , Matsushita, Y. , Ozawa, N. , Ashino, R. , Nakata, M. , … Kawamura, S. (2014). Evolutionary renovation of L/M opsin polymorphism confers a fruit discrimination advantage to ateline New World monkeys. Molecular Ecology, 23, 1799–1812. 24612406 10.1111/mec.12703 PMC4260670

[ece32716-bib-0043] Matsushita, Y. , Oota, H. , Welker, B. J. , Pavelka, M. S. , & Kawamura, S. (2014). Color vision variation as evidenced by hybrid L/M opsin genes in wild populations of trichromatic Alouatta New World monkeys. International Journal of Primatology, 35, 71–87. 24523565 10.1007/s10764-013-9705-9 PMC3915081

[ece32716-bib-0044] Melin, A. D. , Fedigan, L. M. , Hiramatsu, C. , Hiwatashi, T. , Parr, N. , & Kawamura, S. (2009). Fig foraging by dichromatic and trichromatic *Cebus capucinus* in a tropical dry forest. International Journal of Primatology, 30, 753–775.

[ece32716-bib-0045] Melin, A. D. , Fedigan, L. M. , Hiramatsu, C. , Sendall, C. L. , & Kawamura, S. (2007). Effects of colour vision phenotype on insect capture by a free‐ranging population of white‐faced capuchins, *Cebus capucinus* . Animal Behaviour, 73, 205–214.

[ece32716-bib-0046] Melin, A. D. , Fedigan, L. M. , Young, H. C. , & Kawamura, S. (2010). Can color vision variation explain sex differences in invertebrate foraging by capuchin monkeys. Current Zoology, 56, 300–312.

[ece32716-bib-0047] Melin, A. D. , Hiramatsu, C. , Parr, N. A. , Matsushita, Y. , Kawamura, S. , & Fedigan, L. M. (2014). The behavioral ecology of color vision: Considering fruit conspicuity, detection distance and dietary importance. International Journal of Primatology, 35, 258–287.

[ece32716-bib-0048] Melin, A. D. , Young, H. C. , Mosdossy, K. N. , & Fedigan, L. M. (2014). Seasonality, extractive foraging and the evolution of primate sensorimotor intelligence. Journal of Human Evolution, 71, 77–86. 24636732 10.1016/j.jhevol.2014.02.009

[ece32716-bib-0049] Milton, K. (1979). Factors influencing leaf choice by howler monkeys: A test of some hypotheses of food selection by generalist herbivores. The American Naturalist, 114, 362–378.

[ece32716-bib-0050] Milton, K. (1980). The foraging strategy of howler monkeys: A study in primate economics/katharine milton. New York: Columbia University Press.

[ece32716-bib-0051] Milton, K. (1991). Leaf change and fruit production in six neotropical Moraceae species. Journal of Ecology, 79, 1–26.

[ece32716-bib-0052] Milton, K. , & McBee, R. H. (1983). Rates of fermentative digestion in the howler monkey, *Alouatta palliata* (primates: ceboidea). Comparative Biochemistry and Physiology A, Comparative Physiology, 74, 29–31. 6130881 10.1016/0300-9629(83)90706-5

[ece32716-bib-0053] Mollon, J. D. (1989). ‘Tho’ she kneel'd in that place where they grew..’ The uses and origins of primate colour vision. The Journal of Experimental Biology, 146, 21–38. 2689563 10.1242/jeb.146.1.21

[ece32716-bib-0054] Muniz, J. A. P. C. , de Athaide, L. M. , Gomes, B. D. , Finlay, B. L. , & Silveira, L. C. de L. (2014). Ganglion Cell and Displaced Amacrine Cell Density Distribution in the Retina of the Howler Monkey (*Alouatta caraya*). PLoS ONE, 9, e115291. doi:10.1371/journal.pone.0115291. 25546077 10.1371/journal.pone.0115291 PMC4278902

[ece32716-bib-0055] Muñoz, D. , Estrada, A. , Naranjo, E. , & Ochoa, S. (2006). Foraging ecology of howler monkeys in a cacao (*Theobroma cacao*) plantation in Comalcalco, Mexico. American Journal of Primatology, 68, 127–142. 16429417 10.1002/ajp.20211

[ece32716-bib-0056] Osorio, D. , Smith, A. C. , Vorobyev, M. , Buchanan‐Smith, H. M. , & Ryan, A. E. M. J. (2004). Detection of fruit and the selection of primate visual pigments for color vision. The American Naturalist, 164, 696–708. 10.1086/425332 29641923

[ece32716-bib-0057] Osorio, D. , & Vorobyev, M. (1996). Colour vision as an adaptation to frugivory in primates. Proceedings of the Royal Society B: Biological Sciences, 263, 593–599. 8677259 10.1098/rspb.1996.0089

[ece32716-bib-0058] Osorio, D. , & Vorobyev, M. (2008). A review of the evolution of animal colour vision and visual communication signals. Vision Research, 48, 2042–2051. 18627773 10.1016/j.visres.2008.06.018

[ece32716-bib-0059] Parr, N. A. , Melin, A. D. , Fedigan, L. M. , Parr, N. A. , Melin, A. D. , & Fedigan, L. M. (2011). Figs are more than fallback foods: The relationship between ficus and cebus in a tropical dry forest, figs are more than fallback foods: The relationship between ficus and cebus in a tropical dry forest. International Journal of Zoology, International Journal of Zoology, 2011, e967274.

[ece32716-bib-0060] Pereira, R. A. S. , Rodrigues, E. , & de Oliveira Menezes Jr, A. (2006). Phenological patterns of *Ficus citrifolia* (Moraceae) in a seasonal humid‐subtropical region in Southern Brazil. Plant Ecology, 188, 265–275.

[ece32716-bib-0061] Pessoa, D. M. A. , Maia, R. , de Albuquerque Ajuz, R. C. , De Moraes, P. Z. P. M. R. , Spyrides, M. H. C. , & Pessoa, V. F. (2014). The adaptive value of primate color vision for predator detection. American Journal of Primatology, 76, 721–729. 24535839 10.1002/ajp.22264

[ece32716-bib-0062] Regan, B. C. , Julliot, C. , Simmen, B. , Viénot, F. , Charles‐Dominique, P. , & Mollon, J. D. (1998). Frugivory and colour vision in *Alouatta seniculus*, a trichromatic platyrrhine monkey. Vision Research, 38, 3321–3327. 9893844 10.1016/s0042-6989(97)00462-8

[ece32716-bib-0063] Regan, B. C. , Julliot, C. , Simmen, B. , Viénot, F. , Charles‐Dominique, P. , & Mollon, J. D. (2001). Fruits, foliage and the evolution of primate colour vision. Philosophical Transactions of the Royal Society of London B: Biological Sciences, 356, 229–283. 11316480 10.1098/rstb.2000.0773 PMC1088428

[ece32716-bib-0064] Riba‐Hernández, P. , Stoner, K. E. , & Lucas, P. W. (2005). Sugar concentration of fruits and their detection via color in the Central American spider monkey (*Ateles geoffroyi*). American Journal of Primatology, 67, 411–423. 16342072 10.1002/ajp.20196

[ece32716-bib-0065] Rosenberger, A. L. , Halenar, L. , & Cooke, S. B. (2011). The making of platyrrhine semifolivores: models for the evolution of folivory in primates. The Anatomical Record: Advances in Integrative Anatomy and Evolutionary Biology, 294, 2112–2130. 10.1002/ar.21510 22042497

[ece32716-bib-0066] Rothman, J. M. , Raubenheimer, D. , Bryer, M. A. H. , Takahashi, M. , & Gilbert, C. C. (2014). Nutritional contributions of insects to primate diets: Implications for primate evolution. Journal of Human Evolution, 71, 59–69. 24742878 10.1016/j.jhevol.2014.02.016

[ece32716-bib-0067] Salazar, L. T. H. , Dominy, N. J. , & Laska, M. (2015). The sensory systems of Alouatta: Evolution with an eye to ecology In Kowalewski M. M., Garber P. A., Cortés‐Ortiz L., Urbani B., & Youlatos D. (Eds.), Howler monkeys (pp. 317–336). New York: Springer.

[ece32716-bib-0068] Serio‐Silva, J. C. , Rico‐Gray, V. , Hernández‐Salazar, L. T. , & Espinosa‐Gómez, R. (2002). The role of Ficus (Moraceae) in the diet and nutrition of a troop of Mexican howler monkeys, *Alouatta palliata mexicana*, Released on an Island in Southern Veracruz, Mexico. Journal of Tropical Ecology, 18, 913–928.

[ece32716-bib-0069] Setchell, J. M. , Wickings, E. J. , & Knapp, L. A. (2006). Signal content of red facial coloration in female mandrills (*Mandrillus sphinx*). Proceedings of the Royal Society of London B: Biological Sciences, 273, 2395–2400. 10.1098/rspb.2006.3573 PMC1636084 16928644

[ece32716-bib-0070] Smith, A. C. , Surridge, A. K. , Prescott, M. J. , Osorio, D. , Mundy, N. I. , & Buchanan‐Smith, H. M. (2012). Effect of colour vision status on insect prey capture efficiency of captive and wild tamarins (*Saguinus* spp.). Animal Behaviour, 83, 479–486.

[ece32716-bib-0071] Sperling, H. G. , & Harwerth, R. S. (1971). Red‐green cone interactions in the increment‐threshold spectral sensitivity of primates. Science (New York, N.Y.), 172, 180–184. 10.1126/science.172.3979.180 4993975

[ece32716-bib-0072] Sumner, P. , & Mollon, J. D. (2000a). Chromaticity as a signal of ripeness in fruits taken by primates. The Journal of Experimental Biology, 203, 1987–2000. 10851116 10.1242/jeb.203.13.1987

[ece32716-bib-0073] Sumner, P. , & Mollon, J. D. (2000b). Catarrhine photopigments are optimized for detecting targets against a foliage background. The Journal of Experimental Biology, 203, 1963–1986. 10851115 10.1242/jeb.203.13.1963

[ece32716-bib-0074] Valenta, K. , Edwards, M. , Rafaliarison, R. R. , Johnson, S. E. , Holmes, S. M. , Brown, K. A. , … Melin, A. D. (2015). Visual ecology of true lemurs suggests a cathemeral origin for the primate cone opsin polymorphism. Functional Ecology, 30, 932–942.

[ece32716-bib-0075] Valenta, K. , & Melin, A. (2012). Protein limitation explains variation in primate colour vision phenotypes: A unified model for the evolution of primate trichromatic vision. Zoology, http://www.intechopen.com/books/zoology/protein-limitation-explains-variation-in-primate-colorvision-phenotypes-a-unified-model-for-the-evo.

[ece32716-bib-0076] Veilleux, C. C. , & Bolnick, D. A. (2009). Opsin gene polymorphism predicts trichromacy in a cathemeral lemur. American Journal of Primatology, 71, 86–90. 18837042 10.1002/ajp.20621

[ece32716-bib-0077] Welker, B. (2004). Proximate mechanisms governing feeding behavior and selectivity in mantled howler monkeys. Alouatta Palliata: State University of New York at Buffalo.

[ece32716-bib-0078] Yang, D. , Peng, Y. , Zhang, G. , Song, Q. , Zhao, T. , & Wang, Q. (2002). Relationship between population variation of fig trees and environment in the tropical rainforests of Xishuangbanna. Huan Jing Ke Xue= Huanjing Kexue/[bian Ji, Zhongguo Ke Xue Yuan Huan Jing Ke Xue Wei Yuan Hui ‘Huan Jing Ke Xue’ Bian Ji Wei Yuan Hui.], 23, 29–35. 12533922

[ece32716-bib-0079] Yokoyama, S. , & Radlwimmer, F. B. (1998). The ‘five‐sites’ rule and the evolution of red and green color vision in mammals. Molecular Biology and Evolution, 15, 560–567. 9580985 10.1093/oxfordjournals.molbev.a025956

[ece32716-bib-0080] Yokoyama, S. , Yang, H. , & Starmer, W. T. (2008). Molecular basis of spectral tuning in the red‐ and green‐sensitive (M/LWS) pigments in vertebrates. Genetics, 179, 2037–2043. 18660543 10.1534/genetics.108.090449 PMC2516078

